# Transformation of BCC and B2 High Temperature Phases to HCP and Orthorhombic Structures in the Ti-Al-Nb System. Part I: Microstructural Predictions Based on a Subgroup Relation Between Phases

**DOI:** 10.6028/jres.098.038

**Published:** 1993

**Authors:** L. A. Bendersky, A. Roytburd, W. J. Boettinger

**Affiliations:** National Institute of Standards and Technology, Gaithersburg, MD 20899-0001; Dept. of Materials and Nuclear Engineering, University of Maryland, College Park, MD 20742; National Institute of Standards and Technology, Gaithersburg, MD 20899-0001

**Keywords:** domain structure, elastic accommodation, phases, space group relations, structural relations, Ti-Al-Nb system, transformation path

## Abstract

Possible paths for the constant composition coherent transformation of BCC or B2 high temperature phases to low temperature HCP or Orthorhombic phases in the Ti-Al-Nb system are analyzed using a sequence of ciystallographic structural relationships developed from subgroup symmetry relations. Symmetry elements lost in each step of the sequence determine the possibilities for variants of the low symmetry phase and domains that can be present in the microstructure. The orientation of interdomain interfaces is determined by requiring the existence of a strain-free interface between the domains. Polydomain structures are also determined that minimize elastic energy. Microstructural predictions are made for comparison to experimental results given by Benderslcy and Boettinger [J. Res. Natl. Inst. Stand. Technol. 98, 585 (1993)].

## 1. Introduction

The need for low density structural materials with high temperature strength and low temperature ductility has stimulated much interest in Ti-Al-Nb alloys. In particular alloys near and in the Ti_3_Al-Nb_3_Al pseudobinary section with Nb levels from 10 to 30 at% have been investigated [1–8]. It has been shown that alloys with 10–12 and 25 at% Nb have very promising combinations of specific strength and rupture life at room and high (<800°C) temperatures [1,6,9–14]. The mechanical properties of these alloys were found to be very sensitive to their microstructure. Most of the microstructures were formed by heat treatments that involve continuous cooling from a high temperature (> 1100 °C) single-phase field with subsequent heat treatment at lower temperatures. The microstructure developed during continuous cooling depends strongly on cooling rate and alloy composition [15–19] and thus affects the microstructure produced from it during the subsequent lower temperature (600 to 900 °C) treatment. From a technological point of view, an understanding of the formation mechanisms of both continuously cooled and annealed microstructures is very important for processing these alloys for optimum properties, for controlling behavior during thermal cycling, and for obtaining weldability.

Equilibria along the Ti_3_Al-Nb_3_Al pseudobinary section with Nb<30 at% involves phases based on two distinct fundamental structures: body centered cubic (BCC) at high temperatures and hexagonal close-packed (HCP) at lower temperatures. The BCC-based phases appear over a wide range of compositions at high temperatures as either a disordered A2 or an ordered B2 (CsCl-type) structure depending on the exact temperature and composition [2–5,20]. For Ti-Al-Nb alloys the 32 ordering appears to have Ti on one site and Al/Nb on the other site [21]. For low Nb content, the HCP-based structures have a disordered hexagonal *α* phase at intermediate temperatures and an ordered hexagonal DO_19_ phase (Ti_3_Al or α_2_) at low temperatures. At higher Nb contents, the HCP-based low temperature structure is an orthorhombic O-phase (Ti_2_AlNb) [2,4–6,22]. The DO_19_ structure involves binary ordering of the a structure with Ti/Nb occupying one site and Al the other site [23]. The O-phase structure involves further ternary ordering of the α_2_ phase with Ti, Al, and Nb predominantly occupying three different sites [2,24]. Due to the structural relations (reflected in the observed Burger’s orientation relationships between the phases [2–4,7,8,25]) it is well known that the structural changes (transformations) from BCC-to HCP-based phases can be described as a result of the following, where (*hkl*)_c_ refers to the cubic phase:

1. distortions of {110}_c_ planes and changes in their interplanar distances;
2. shuffles, or relative displacement of neighboring (110)_c_ planes;
3. reordering that changes the distribution (occupancy) of Ti, Al and Nb atoms among the lattice sites.

In the spirit of the Landau theory of phase transitions [26] a common framework is sought to describe all of the BCC- and HCP-based phases. Then in principle, a single thermodynamic potential can be identified as a continuous function of a set of order parameters that describe these three types of structural changes. To obtain such a common framework, a site-to-site correspondence between the structures must be found. This correspondence between atom sites in these phases can be obtained by a single set of Wyckoff sites of the lowest symmetry phase considered, which in this case is the orthorhombic O-phase. Changes in the coordinates and occupancies of the Wyckoff positions are related to the three types of structural changes mentioned above. Special values of the site occupancy parameters (and lattice parameters) will correspond to changes in crystal symmetry that will follow subgroup/supergroup paths. Analysis of these paths lead to important information regarding the sequence of phase transitions, possible transient states, interconnections between the structures, and domain interface configurations.

To make this approach valid, two assumptions are required:

1. The transformations are diffusionless, i.e., no changes in compositions of phases may occur. The assumption seems to be valid considering the time scale of long-range diffusion as compared the interatomic jumps or displacements required for chemical or displacive ordering respectively;
2. The transformations are coherent, i.e., no discontinuities occur by slip or fracture in order to relieve internal stress during the phase transformation.

Both assumptions are likely to hold for the Ti-Al-Nb compositions considered either during sufficiently fast continuous cooling from the high temperature single-phase BCC or B2 field or during the initial stages of isothermal annealing of the metastable quenched-in phase. Experimental microstructural results indicate the occurrence of martensite-type ordering transitions and coherent structures under these conditions. Phase separation involving long-range compositional diffusion will be treated by Bendersky and Boettinger [J. Res. Natl. Inst. Stand. Technol. **98**, 585 (1993)].

In this paper we analyze the possible continuous transformations on cooling in the Ti-Al-Nb system and the expected features of idealized coherent microstructures. The approach here is to see the transformations as a sequence of symmetry reductions, and microstructure as a collection of domains. The microstructural features determined by this analysis will be used for comparison to the actual experimental results of Bendersky and Boettinger based on microstructural studies of three Ti-Al-Nb alloys, mainly by transmission electron microscopy (TEM).

Prediction of the microstructural features relies almost entirely on the known structural and symmetry relations between the highest and lowest symmetry phases. The necessary information concerning the symmetry relations is contained in the space group tables of the International Tables for Crystallography [27]. Based on this information, maximal group/subgroup symmetry relations between phases will be established in Sec. 2. Each transformation step will be considered as a symmetry change, and the transformation path as sequence of subgroups. The symmetry analysis can preclude certain transformation paths, assist in an interpretation of the observed paths and also predict possible intermediate phases. From the predicted path, domain structures can be anticipated. Such domain structures will consist of a hierarchical distribution of interfaces due to the formation of orientational (twin) and translational (anti-phase domain) variants, (Sec. 3). It is expected that the formation of domain structures will minimize the elastic energy arises due to the coherency of transformation. Therefore, low energy, stress free interfaces (SFI) between orientational domains as well as their mutual arrangement are considered in Sec. 4. In Sec. 5 results from the previous sections will be summarized to show what microstructures are expected to be seen for different transformation paths.

## 2. Group/Subgroup Relations Between BCC $\left(Im\bar{3}m\right)$, HCP (P6_3_/mmc) and Ordered Orthorhombic (Cmcm) Phases

### 2.1 Sequence of Maxima) Subgroups

The Landau theory of phase transition of first or higher order assumes that the symmetry of the product phase is a subgroup of the parent phase and that the atomic positions of the two structures are closely related by a set of order parameters. Usually the low temperature phase has symmetry lower than the high temperature phase and the decrease in symmetry is known as ordering while an increase in symmetry is known as disordering. The group/subgroup relationship between the parent and product phases need not be maximal.^1^ However in this paper, we will search for a sequence of maximal group/subgroup relationships in order to anticipate all possible (but not necessarily occurring) intermediate states. Such a sequence can be obtained using the International Tables for Crystallography [27], where the maximal subgroups and supergroups of all 230 crystallographic space groups are tabulated. Table 1 gives examples of such subgroup tables for (a) the Im3¯m space group (e.g., of the BCC structure) and (b) the P6_3_/mmc space group (e.g., of the HCP structure). Examples of the known structures represented by these subgroups are B2 $\left(\mathrm{Pm}\bar{3}m\right)$ in the IIa subgroups of Im3¯m and DO_19_ (P6_3_/mmc) in the IIc subgroups of P6_3_/mmc.

Often there is no apparent subgroup relation between parent and product phases. Examples are found for transitions between structures with cubic and hexagonal symmetry, like the phases in the Ti-Al-Nb system. Here the non-coinciding 3-fold 〈111〉 cubic and 6-fold [0001] hexagonal symmetry axes preclude such a relation. Usually transformations between two phases which do not have a group/subgroup relation are considered reconstructive and not treated by the Landau approach. A connection between symmetries can be restored in some cases by introducing an intermediate structure with space group *G*_t_ that is either a supergroup of both structures, *G*_1_ and *G*_2_, or a subgroup of both structures [28]. When *G*_1_ is a supergroup (also called a paraphase in [28]), it is at least a group union of the *G*_1_ and *G*_2_ groups and might not necessarily exist. Such is the case for the BCC and HCP phases considered here which already have very high symmetry. However a subgroup, *G*_1_ can always be found (and not necessarily the trivial group P1) as the intersection group of *G*_1_ and *G*_2_. In particular, for the disordered BCC and HCP phases with Im3¯m and P6_3_/mmc space groups and an orientation of unit cell axes according to the Burger’s relationship (parallel close packed directions $[111{]}_{c}//[11\bar{2}0{]}_{h}$ and planes (111)_c_//(0001)_h_), the intersection group *G*_t_ is the orthorhombic Cmcm, with its *c*-axis parallel to the [110]c direction.

**Fig. 1:**
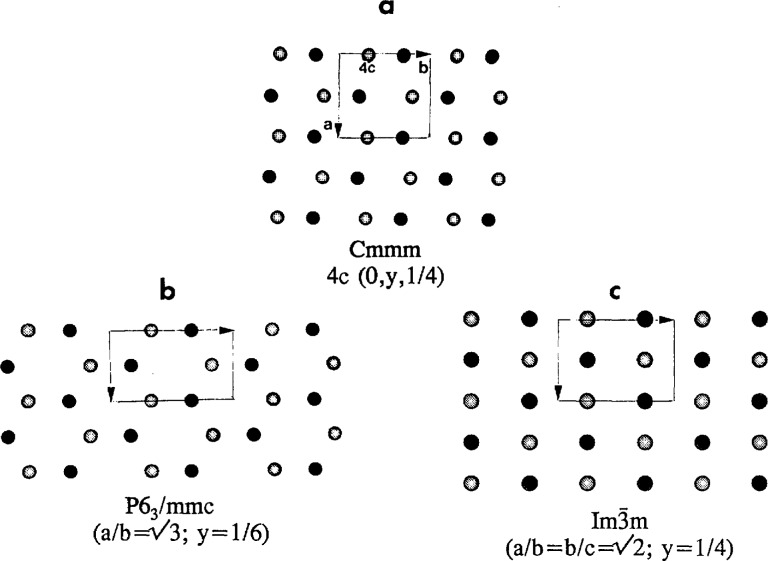
The Cmcm space group is represented by the structure (a) which is close to both the HCP (b) and BCC (c) but nevertheless different in symmetry and the relative positions of their basal planes. The structures are shown in projections along their [001] (*a,b*) and [110] (c) directions. Black and white shades represent two neighboring layers of atoms.

The Cmcm space group (with appropriate choice of Wyckoff sites) can represent a structure which is close to HCP but differs in symmetry and relative position of the atoms in the basal planes (Fig. 1). Such an intermediate structure was reported as a martensitic phase in some Ti alloys [29]. The Cmcm structure can also be considered as the BCC structure distorted by shuffles (relative shifts) of the (110)_c_ planes. In this case the Cmcm group could have been found procedurally by taking the intersection of the cubic symmetry and the symmetry of the shuffle displacement wave (mmm point group symmetry for the $\left(110)[\bar{1}10\right]$-type shuffle [30,31]). Such symmetry can be locally present in the premartensitic tweed states of quenched BCC or BCC-based structures, which are also known to have the $\left(110)[\bar{1}10\right]$-type soft phonon modes (tweed BCC).^2^

**Fig. 2:**
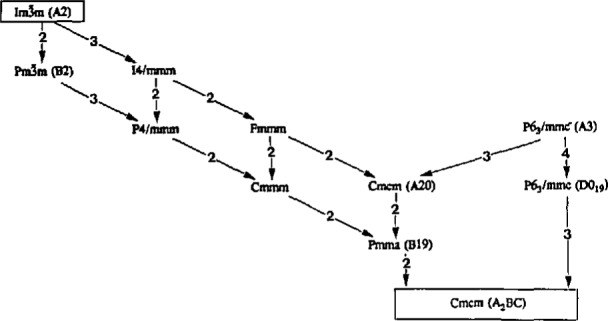
Subgroup/supergroup symmetry relations between the high symmetry Im3¯m (BCC) and the lower symmetry orthorhombic Cmcm (Ti_2_AlNb) space groups. Space groups are connected to each other with arrows pointing in the direction of a decrease in symmetry. The number shown in square brackets next to each arrow is the index of symmetry reduction. Vertical arrows are used to indicate changes in symmetry due to displacive ordering. Angled arrows indicate that the difference in symmetry is due to changes in atomic site occupancy (chemical ordering).

Sequences of maximal subgroups were found that connect the highest symmetry cubic and hexagonal space groups to the low symmetry orthorhombic “intersection” space group (Fig. 2). This sequence includes all known equilibrium phases observed in alloys near the Ti_3_Al-Nb_3_Al section with less than 30 at% Nb. The figure includes sequences along disordered (BCC) and ordered (B2) branches of the high temperature phases. In the figure the space groups are connected to each other with arrows indicating *symmetry decrease.* The numbers shown are the indices of symmetry reductions between two neigh-boring subgroups (the index of a subgroup is the ratio of the number of symmetry elements in a group to that of the subgroup). These integers give the number of lower symmetry variants (domains) that would be possible if a transition from high to the low symmetry occurred. Inclined arrows indicate symmetry changes due to atomic site (Wyckoff) position changes leaving the occupancy fixed, i.e., displacive ordering. Vertical arrows indicate symmetry changes due primarily to changes in atomic site occupancy, i.e., chemical ordering. Slight adjustments of site positions and occupancies due to the new atomic environments will accompany the chemical and displacive ordering respectively. As described in Sec. 3, one possible transformation sequence for the formation of the lowest symmetry orthorhombic phase from BCC will involve symmetry increase (supergroup formation) from the A20 structure to the A3 structure. In this case no new variants are formed.

### 2.2 Intermediate Subgroups and Their Corresponding Structures

**Fig. 3:**
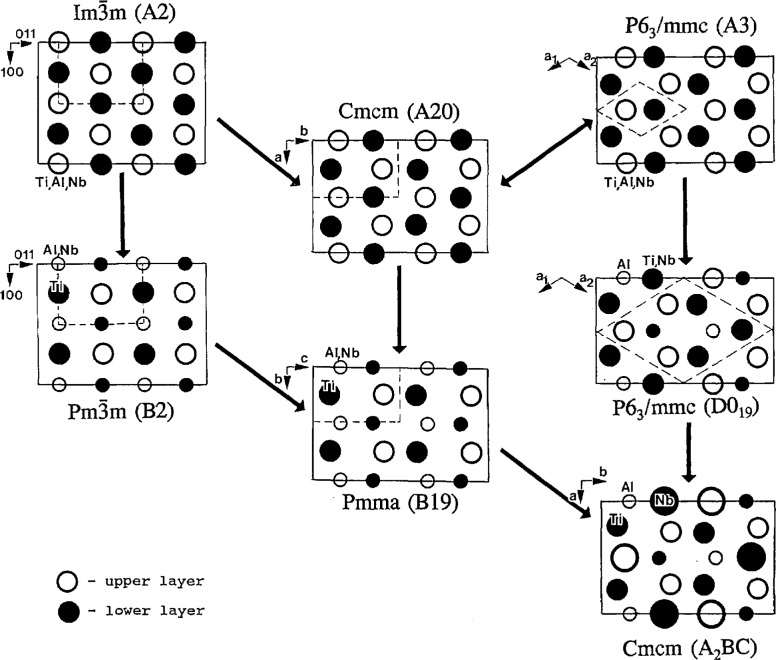
Structures corresponding to the Fig, 2 subgroup sequence, as viewed along the [001]_u_ ([011]_c_) direction. Frames of the largest unit cell (of the O-phase) and of each particular crystal structure are drawn. Increasing size circles represents Al, Ti, and Nb atoms, respectively. Filled and empty circles correspond to different parallel layers of atoms.

**Fig. 4:**
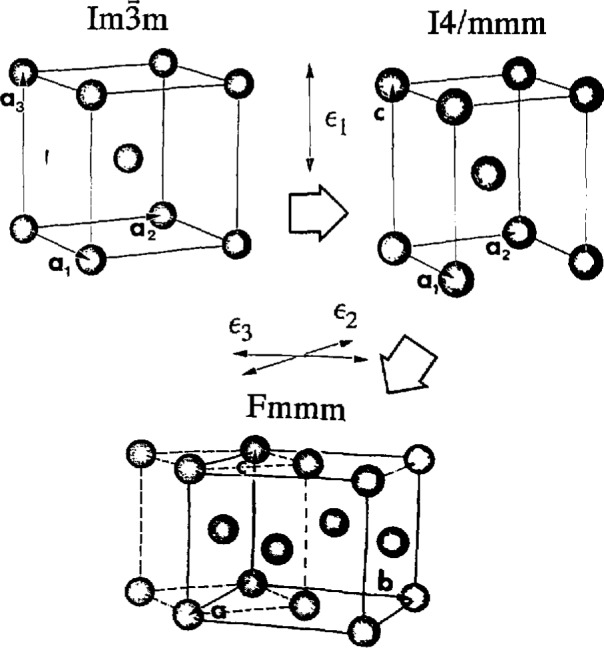
Space groups and structures representing homogeneous strain distortion of the cubic lattice (a), (b) The I4/mmm and P4/mmm are structures of tetragonal distortion along cubic 〈100〉 (with Wyckoff positions 2a and 1a, 1d, respectively). (c) The Fmmm and Cmmm are structures with orthorhombic biaxial distortion along orthogonal cubic 〈011〉 directions (with Wyckoff positions 4a and 2a,2c, respectively, and a doubled size unit cell.

In addition to the space groups of the Ti-Al-Nb equilibrium phases, A2(BCC): Im3¯m, B2: $\mathrm{Pm}\bar{3}m$, A3(HCP):P6_3_/mmc, DO_19_:P6_3_/mmc and O-phase (Ti_2_AlNb:Cmcm), several other space groups must be introduced in order to keep the subgroup relation maximal (Fig. 2). Crystallographic structures corresponding to the space groups in Fig. 2 are shown in Figs. 3 and 4 (with occupancies relevant for the ternary Ti-Al-Nb alloys). These figures assume an atom to atom correspondence between the structures. In Fig. 3 all of the structures are presented in a common projection normal to their close-packed planes. Frames of both the largest unit cell (of the O-phase) and of the particular crystal structure unit cells are also shown. Analysis of the intermediate structures lead to the following details.

The I4/mmm and Fmmm_structures (obtained from the disordered BCC, Im3¯m) and the P4/mmm and Cmmm structures (obtained from the ordered B2, $\mathrm{Pm}\bar{3}m$) represent homogeneous strain of the cubic lattice. The I4/mmm (Wyckoff position 2a) and P4/mmm (Wyckoff positions la and Id) are tetragonally distorted along the cubic 〈100) direction. The Fmmm and Cmmm are structures with different distortions along two orthogonal cubic 〈001) directions (with Wyckoff positions 4a and 2a, 2c, respectively, and a doubled unit cell, recentered and rotated by 45° (***a***′=***a***+***b***; ***b***′=***a***−***b***). The homogeneous strains of orthorhombic symmetry do not change the number of atoms per primitive cell–there remains one atom/cell for the disordered and two atoms/cell for the ordered structures (Fig. 4).

The overall orthorhombic distortion of the cubic structure, if not supported by ordering, is most probably unstable for materials with simple metallic bonding. Therefore, these structures are not expected to exist as metastable states but rather represent a homogeneous strain accompanying (and selecting the orientation of) the subsequent symmetry reduction by shuffle displacement (from Fmmm and Cmmm to Cmcm and Pmma, respectively).

**Fig. 5:**
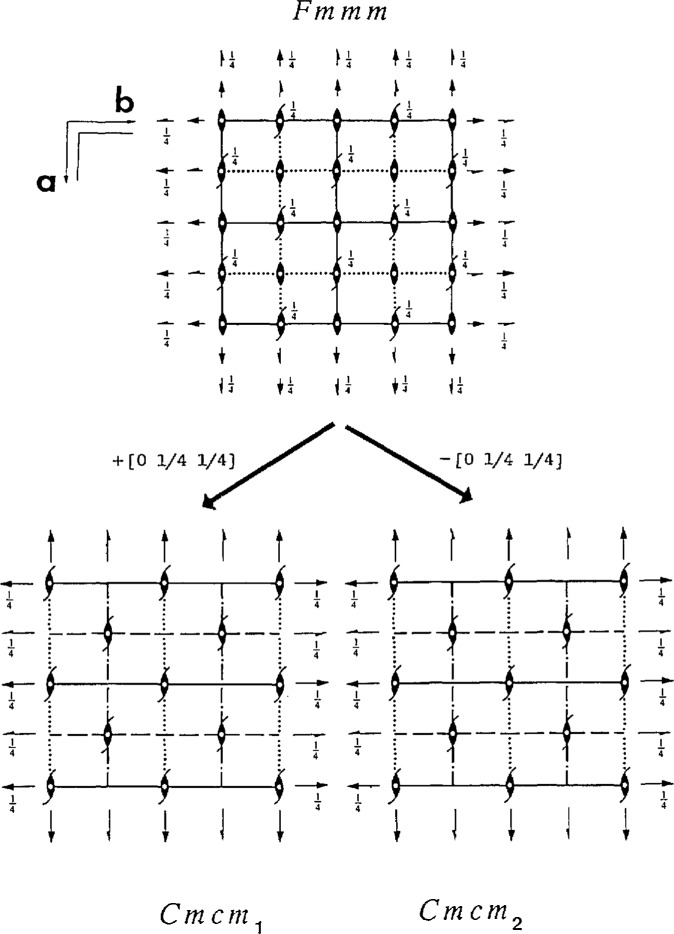
Comparison of the space-group diagrams of Fmmm and two translational variants of Cmcm. In order to have coincidence of common symmetry elements of these two space groups, the Cmcm diagrams must have a coordinate origin shifted to either 0,–1/4,–1/4 or 0,1/4,1/4. The translation vector between two Cmcm variants is [0 1/2 1/2].

Structures corresponding to the Cmcm and Pmma space groups are known in the literature as the Struckturbericht A20 (α-U prototype) and B19 (AuCd prototype), respectively. These structures can be obtained by heterogeneous shuffles of pairs of (110)_c_ planes of either disordered or ordered cubic structure [corresponding to either 100 or 010 planes of the Fmmm and Cmmm, respectively (Fig. 4)]. The amplitude of the shuffle displacement wave is reflected in the parameters of the *y* coordinate of Wyckoff positions: 4c (0,*y*,1/4) for the Cmcm structure and 2e (l/4,*y*_1_,0); 2f (1/4,*y*_2_,1/2) for Pmam. The symmetry changes do not depend on the size of the displacement. The effect of the shuffles on the disordered Fmmm (010) is that its mirror planes are changed into diagonal glide planes, and all two-fold axes disappear, as shown in Fig. 5 which compares the symmetry elements of these two space groups. The Cmcm structure (or equivalently Amam for the Fmmm coordinate system) has a shifted coordinate origin at either 0, – 1/4, – 1/4 or 0,1/4,1/4 (in order to have coincidence of common symmetry elements as shown in Fig. 5). Similarly, for the ordered Cmmm (010) mirror planes and two-fold axes disappear, and the symmetry became Pmam (or conventional Pmma [27] with a permutation of the ***b*** and ***c*** axes). A new coordinate origin of the Pmma is also at ± 1/4,0,1/4.

The two origins correspond to two translational variants, with a (0,1/2,0) displacement vector. Formally, from the maximal subgroup relations [27], the translational variants are the result of a lattice decentering. Structurally, the formation of the two variants can be described by shuffle displacement waves that are out of phase by a half period in opposite directions. Because of the displacive nature of ordering, a translational interface between them has a stacking fault nature with atomic distances different from the bulk material. We will discuss details of the interface structure later.

For some special values of Wyckoff positions (*y* coordinates) and/or of lattice parameters, a structure can degenerate into a structure of higher symmetry. Such a higher symmetry structure, the hexagonal P6_3_/mmc (Fig. 3), occurs for the disordered Cmcm when the shuffles are such that Wyckoff position parameter *y* is 1/3 and the ratio of lattice parameters, *b*/*a*, is $\sqrt{3}$. For the ordered orthorhombic Pmma such a symmetry increase by displacement is precluded by the chemical order inherited from the B2. The disordered hexagonal P6_3_/mmc is expected to be more stable than the disordered orthorhombic Cmcm; in a hard sphere approximation P6_3_/mmc has higher entropy (due to its higher symmetry) while interaction energies are comparable. No thermodynamic barrier for the Cmcm (A20) to P6_3_/mmc (A3) transition is expected, and therefore the disordered Cmcm structure is believed to be unstable. This conclusion cast doubts on the existence of the truly disordered orthorhombic martensite [29]. (Nevertheless disordered Cmcm(A20) structures are known for U, Am, Ce, Ga with nonspherical electron densities). Conversely, the Pmma structure (B19) could well be a stable or a metastable phase and exist as a transient state. Indeed numerous B19 phases are known in different systems as either metastable (martensitic) or stable phases, e.g., AuCd and NiTi.

The structure with the lowest symmetry, the O-phase, also has the Cmcm space group and ternary ordering on three Wyckoff positions, 4ci, 4c2, and 8g. The O-phase translations on basal (001)_o_ plane are twice that of the binary ordered Pmma (B19). The structure can be obtained by ordering either Pmma (B19) or P6_3_/mmc (DO_19_). The DO_19_ structure itself can be obtained by binary ordering of the disordered HCP (A3) and could be in a intermediate metastable state.

### 2.3 Description of Structures as Special Cases of the Lowest Symmetry Cmcm

To summarize, the structures from the group/subgroup sequence can be described in terms of the lowest symmetry Cmcm space group corresponding to the Ti_2_AlNb phase. The orthorhombic Structure has three Wyckoff positions, 4c_1_ {0,*y*_1_, 1/4), 4c_2_ (0,*y*_2_,l/4) and 8g (*x*_3_,*x*_3_,l/4). Special values of the Wyckoff coordinates, the site occupancies. and the ratios of the orthorhombic lattice parameters can describe all the structures. The results are summarized in Table 2 where the space groups, Struckturbericht name, prototypes, restrictions on lattice parameters (if any), occupancies (general values and measured values for Ti,Al,Nb) and coordinates of the Wyckoff sites are presented. From the schematic representation of the data in Fig. 3 one can visualize the transformation sequence as a continuous change of atomic sites occupancies and positions within the framework of the O-phase. Regardless of whether the transformations occur by a continuous mechanism, the common geometrical description of the known equilibrium phases permits the realization, in principle, of a single thermodynamic potential representing all of the phases as a function of a set of order parameters based on the site positions and occupancies.

## 3. Transformation Paths, Types and Hierarchy of Domains Interfaces

The formal crystallographlc sequence of group/subgroup relations (Figs. 2 and 3) suggests different ways that the coherent phase transformations from the high temperature BCC (Im3¯m) phase might occur in reality. While the direct formation of the lowest symmetry phase by a reconstructive transformation is possible, microstructural evidence presented by Bendersky and Boettinger suggests the contrary. The transformation proceeds by steps according to the sequence which imply metastable transient phases. Each transient phase may exist over some temperature interval between upper (to the supergroup phase) and lower (to the subgroup phase) critical temperatures (or temperatures of phase instability for 1st order transitions). Each transformation step of the sequence will reduce a crystal of the higher symmetry phase into lower symmetry phase variants (except A20 → A3 where an increase of symmetry does not lead to new variants). The orientation and relative translation of the variants will be related to each other by the symmetry operations of the preceding higher symmetry phase that disappeared after the transition. Therefore a hierarchical (in a sense of both symmetry reduction and domain interface distribution) microstructure is expected. Assuming the nucleation of different low symmetry phase variants in each variant of the high symmetry phase and the absence of significant domain coarsening, a hierarchy of microstructural scale is also expected. The transformation sequence can then be recognized by the way in which the variants of the lowest symmetry phase are grouped.

Starting from the A2 (BCC) phase, the lowest symmetry O-phase can be obtained along different transformation paths (different sequences of transformation steps). Using the maximal subgroups relations in Fig. 2 and reasoning about the stability of structures discussed in Sec. 2.2, one finds that following three transformation paths are feasible:

Path1:Im3¯m(A2)→[12]→Cmcm(A20)→[1]→P63/mmc(A3)→[4]→P63/mmc(DO19)→[3]→Cmcm(O)Path2:Im3¯m(A2)→[2]→Pm3¯m(B2)→[12]→Pmma(B19)→[2]→Cmcm(O)Path3:Im3¯m(A2)→[12]→Cmcm(A20)→[2]→Pmma(B19)→[2]→Cmcm(O)

where the numbers in brackets are the number of variants possible after symmetry the change.

The microstructures resulting from these sequences will consist of the same O-phase but with distinctly different hierarchies and types of interfaces. The type of interfaces, either rotational, translational or mixed, is obvious from the group/subgroup relation. Each symmetry reduction has necessarily more then one variant of the low symmetry phase. Variant generating operations and their matrices, *g_ij_*, can be obtained with the help of the International Tables for Crystallography [27] from the list of *Symmetry Operations* of the space group, after excluding the symmetry operations of the subgroup listed in the *Maximal subgroups* table (see Table 1).

The number of variants in each transition is equal to the index of the subgroup (square brackets in Fig. 2 and in paths 1–3). For a sequence of transitions the number of lowest symmetry phase variants (with respect to the highest symmetry phase) will be the product of indices for each step. For the transformation, paths 2 and 3, the number of variants is the same; viz., 48 (2×12×2 or 12×2×2). For the transformation path 1 more variants occur; viz., 144 (12×1×4×3) because of the hexagonal symmetry present as an intermediate state. The index of 12 in paths 1–3 is the product (3 × 2 × 2) of indices of the individual maximal subgroups that accomplish the homogeneous distortion and the shuffles between Im3¯m and Cmcm or between $\mathrm{Pm}\bar{3}m$ and Pmma.

The maximal subgroups in [27] (e.g., shown in Table 1) are divided into isomorphic and non-isomorphic subgroup classes. The isomorphic subgroups (lie) differ from their parent group only by a translation group; i.e., an increased unit cell size (e.g., HCP to DO_19_ ordering which maintains the same rotation group but doubles the unit cell dimensions in the basal plane). The non-isomorphic class is divided into three subclasses. Class IIa, in which the unit cell is decentered, will have translational variants similar to the isomorphic subgroups (e.g., ordering of BCC ( Im3¯m) to B2( $\mathrm{Pm}\bar{3}m$)). Class lib, in which the unit cell is decentered *and* enlarged, will also have only translational variants (e.g., ordering in the Fe-Al system of the B2( $\mathrm{Pm}\bar{3}m$) to the DO_3_ ( $\mathrm{Fm}\bar{3}m$) phase). Therefore variants of these Classes, IIa, IIb and IIc, are purely translational. The third type of non-isomorphic subgroup is Class I (t subgroups), which retain all translation, and have only rotational variants (e.g., transition in the YBa_2_Cu_3_O_7−δ_ high-*T*_c_ superconductor from the tetragonal P4/mmm to the orthorhombic Pmmm superconducting phase). For non-maximal subgroups translational/rotational combinations are possible. In Table 3, the type of interfaces which are created in each group/subgroup transformation step of the transformation paths 1–3 are summarized.

In the case of coherent structure formation, the contacting volumes of the different variants, which form differently oriented or shifted lattices with respect to each other, are known respectively as rotational and translational *domains.* Mixed rotational/translational domains are also possible for transitions with non-maximal subgroup relation. A single rotational variant of a transformation usually has slightly different orientation of axes with respect to its parent than those following the structural correspondence. The orientation depends on the kind of variant of the surrounding domains and the interface orientation. In general, the number and orientation of coexisting domains as well as the configuration of the domain interfaces, i.e., the *domain structure*, depend on the thermodynamics and kinetics of the phase transformation.

Two major factors will effect the morphology and the orientation of equilibrium interfaces–their surface energy and their bulk elastic energy due to the misfit between different variants and between the matrix and different variants. For rotational domains the self-strains generate significant long range strain and one expects elastic energy minimization to dominate the selection of the interface patterns, as described in detail in Sec. 4. For translational domains, (class II subgroup transitions) there is no change of crystal system (e.g., cubic to cubic lattice in the BCC→B2 transition), and therefore only dilatational strains are expected. Thus the surface energy, or more precisely its anisotropy, controls the morphology. However, as many examples from ordered alloys show, these surface energies often have weak anisotropy and domain walls are isotropic and wavy. This is especially true for chemical (substitutional or interstitial) ordering [33]. Based on this, wavy isotropic interfaces are expected for the following transitions presented in Table 3: Im3¯m→Pm3¯m, P6_3_/mmc(A3)→P6_3_/mmc (DO_19_), Cmcm(A20)→Pmma(B19) and Pmma (B19)→Cmcm(O). Less clear are two cases of translational domains in the Im3¯m→Cmcm (A20) and the $\mathrm{Pm}\bar{3}m\to \mathrm{Pmma}$(B19) transitions as discussed in detail in Appendixes A and B.

## 4. Equilibrium Structure of Rotational Domains: Interfaces and Their Arrangement

For rotational domains the elastic energy dominates the interfaciai energy for sufficiently coarse structures. We will only consider equilibrium features of rotational domain structures which minimize elastic energy while ignoring their interfaciai energy. For interfaces with equivalent elastic energy, the interfaciai energy, which can be different for different types and orientations of interfaces (even for the same pair of variants), determines the relative stability.

It is convenient to subdivide the elastic problem into two steps. First, we will consider the simplest domain structure–two domains of two different variants. Secondly, using results for the domain pairs, we will discuss the domain structures consisting of more than two domains. The results, first discussed in general terms, will be applied to two transformations in the Ti-Al-Nb system involving rotational domains: cubic to orthorhombic ( Im3¯m→Cmcm, $\mathrm{Pm}\bar{3}m\to \mathrm{Pmma}$) and hexagonal to orthorhombic (P6_3_/mmc→Cmcm).

### 4.1 Pairs of Domains

The most important characteristic which determines a domain structure is *self-distortion, S_ij_*, or its symmetric part, *self-strain, e_ij_.* The self-strain is a homogeneous macroscopic strain that accompanies each phase transformation. The inhomogeneous strains associated with shuffles can be neglected since their effects cancel over a few atomic dimensions. Different variants of each transformation are characterized by different self-strain tensors according to different orientations of the crystal axes of the variants, and therefore of the principal axes of the tensor. The self-strain tensors of two different variants, e.g., 1 and 2, are connected by the following relation:

$$e_{ij}(2)=g_{ik}g_{jl}e_{kl}(1),$$ (1)

where *g_ik_* is the matrix of one of the parent phase space group symmetry operators which are not a part of the space groups of the two variants. Operating on the self-strain tensor of one variant with these lost symmetry elements generates the self-strain tensors for the other variants. Examples of these matrices for the hex→O-phase and the BCC→O-phase transformations are given in the Appendix A.

In a coherent crystalline system incompatibility of the self-strains on both sides of the interface between domains creates internal stress originating from the interface. Such stress will not arise, and *a stress-free interface* (SFI) will result if

1. the interface is planar, and
2. the self-strains on both sides are compatible, i.e., no discontinuity of displacements occurs at the interface.

To meet the requirement of compatibility it is necessary and sufficient that the difference between two self-strains can be represented as the symmetric part of a diadic product of two unit vectors, ***m*** and ***n***:

$$\Delta e_{ij}=e_{ij}(2)-e_{ij}(1)=\frac{1}{2}s(m_{i}n_{j}+n_{i}m_{j})$$ (2)

where *m_i_* is a vector normal to the planar interface considered, *n_j_* is a vector orthogonal to the *m_i_*, and *s* is a scalar measure of self-strain difference [33]. The rotation of the variants necessary to maintain contact between the domains is given as

$$\omega_{ij}=\pm \frac{1}{2}s(m_{i}n_{j}-n_{i}m_{j}).$$ (3)

**Fig. 6:**
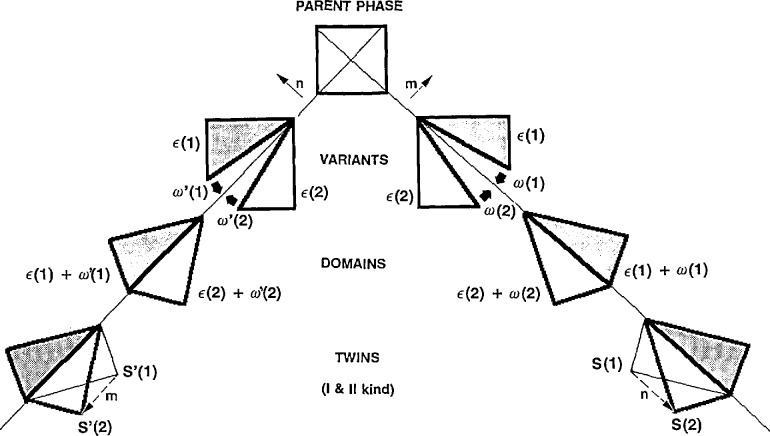
Two-dimensional example of the square to rectangular (p4mm to p2mm) transition illustrating formation of two pairs of domains, their rotations, strain-free interfaces and description by twinning.

When the strain difference given in Eq. (2) is combined with the relative rotation of domains given in Eq. (3), a simple shear will describe the relationship between the two domains. The distortion tensor describing this simple shear is either *sn_i_m_j_* along a plane with ***m*** normal in the direction ***n*** or *sn_i_m_j_* along the plane with ***n*** normal in the direction ***m***. These simple shears are twin shears, and the domains can be considered as twins with two twinning planes, ***m*** *or* ***n***, normal to each other. One of these twinning planes coincides with a mirror plane of the parent crystal structure (which is not a symmetry element of the variants under consideration) and therefore has rational indices. The other one can be a plane with irrational indices in coordinates of the parent crystal. The rational mirror plane corresponds to type I twinning, whereas the second, irrational plane, corresponds to type II twinning [34]. Using a simple two-dimensional example of a square to a rectangle (p4mm to p2mm) transition, Fig. 6 illustrates the operations described above. (Only Type I twinning occur in this example.)

The orientation of SFIs as well as the domain misorientations can be found directly from Eq. (2) in the coordinate system of the principle axes of the strain difference tensor *Δe_ij_* [33,35]. For all other coordinate systems it is convenient to transform Eq. (2) by multiplying it by *x_i_x_j_*, where *x_i_* is an arbitrary vector belonging either to the ***m*** (*x_i_m_i_* = 0) or ***n*** (*x_i_n_i_* =0) SFI planes to obtain [36]

$$x_{i}[e_{ij}(2)-e_{ij}(1)]x_{j}=0.$$ (4)

This quadratic equation splits into the product of two linear equations whose solutions determine the coordinates of two SFI planes. (The absence of a solution of Eq. (4) implies that the difference between the self-strains of the variants can not be represented in the diadic form of Eq. (2), and therefore a domain pair generated by these variants cannot have a SFI.)

Both equivalent equations, Eqs. (2) and (4), were obtained with the assumption that the self-strains are small [33, 36]. However, Eq. (4) can be easily generalized to avoid the small-strain approximation using standard finite deformation analysis. A plane belonging to the parent phase becomes a SFI if any vector *x_k_* in that plane, after being transformed, will have the same length in both variants. In variant (1) the vector *x_k_* becomes *X_i_*(1) = [*δ_ik_+ S_ik_*(1)]*x_k_*, where *δ_ik_* is a unit matrix, *S_ik_*(1) is a self-distortion tensor of the variant 1. The variant (2) transforms the same vector *x_k_* into *X_i_* (2) = [*δ_ik_ + S_ik_* (2)]*x_k_.* The equality of the lengths [*X_i_*^2^(1)=*X_i_*^2^(2)] leads to an equation similar in its form to Eq. (4) but where

$$e_{ij}=\frac{1}{2}(S_{ij}+S_{ji})+\frac{1}{2}S_{ik}S_{kj}$$ (5)

Eq. (5) is a strain tensor commonly used to describe finite deformation and provides an exact definition of self-strain as a symmetric tensor based on the known self-distortion tensor of a transformations, and it includes a quadratic term of the distortion tensor, *S_ij_*. For weakly first order and second order ferroelastic-type transformations, the quadratic term can be neglected in the vicinity of transformation because *S_ij_* (related to the order parameter) is small. For strongly first order martensitic transformations, with large distortions, the quadratic term can be considerable.

**Fig. A.2:**
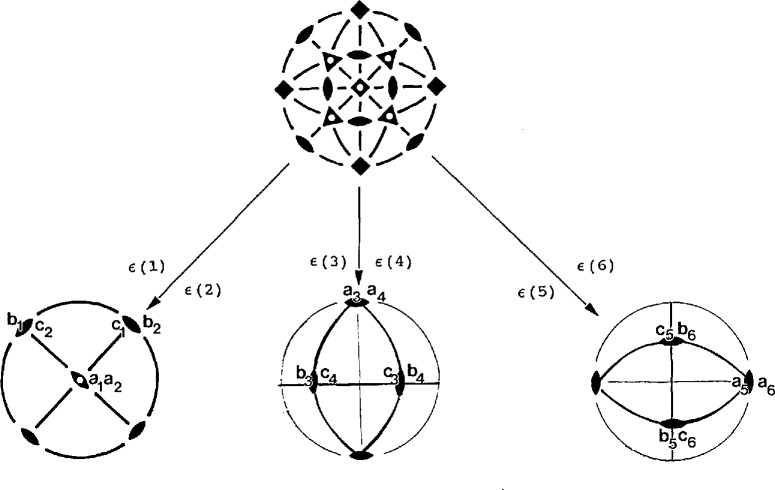
Stereographic projections of the point groups and the crystallograpJiic axes of the parent cubic ( $m\bar{3}m$) and its six subgroup variants of the orthorhombic (mmm) phases. Comparing symmetry elements belonging to a pair of the variants with those of the parent cubic, mirror planes that reflect the variants into each other can be found.

Besides the solutions corresponding to the rational mirror planes in the parent phase, Eq. (4) has solutions that depend on the lattice parameters of the product phases, and therefore yield orientations that are generally irrational and depend on transformation temperature and the phase compositions. The solutions for orientations of SFIs for 94 different combinations of higher and lower point groups, relevant for ferroelastic transformation, are given by Sapriel by solving Eq. (4) [36]. For the transitions considered in this work, namely for BCC/B2 to orthorhombic/HCP structures [ Im3¯m(A2)→Cmcm(A20)/P6_3_/mmc(A3) and $\mathrm{Pm}\bar{3}m$(B2)→Pmma(B19)] and for HCP to orthorhombic [(P6_3_/mmc(DO_19_)→Cmcm(O-phase)], specific forms of Eq. (4) and its solutions are given in Appendix A. For the HCP→orthorhombic transformation, there are only symmetric SFIs of the ${\left\{1\bar{1}00\right\}}_{h}$ and ${\left\{11\bar{2}0\right\}}_{h}$ types (the irrational solution degenerates into a symmetric one). The SFIs correspond to {110}_0_ and {130}_0_, respectively, when transformed to coordinates of the O-phase. For the BCC→orthorhombic (or similarly HCP) transformation, there are three SFIs of the {100}_c_ type, six of the {110}_c_ type, and six irrational {*hhk*}_c_ types with *h*/*k* ratios depending on the lattice parameters of the orthorhombic (or hexagonal) phase. The rational SFIs correspond to {021}_o_ and {221}_o_, respectively, when transformed to coordinates of the O-phase. The {*hhk*}_c_-type interfaces in the O-phase coordinates are of the form {1,{*s*−l),2(s + 1)}_o_ where *s*=*k*/*h.* The {*hhk*}_c_ interfaces, when calculated for the lattice parameters of the DO_19_ or O phases taken from the literature [4], are found to be close to {155}_c_ and {144}_c_, respectively. The pair of orthogonal interfaces between different pairs of variants are summarized in Table 4 where the labeling of the pairs is given according to Fig. A.2.

### 4.2 Polydoitiain Structures

**Fig. 7:**
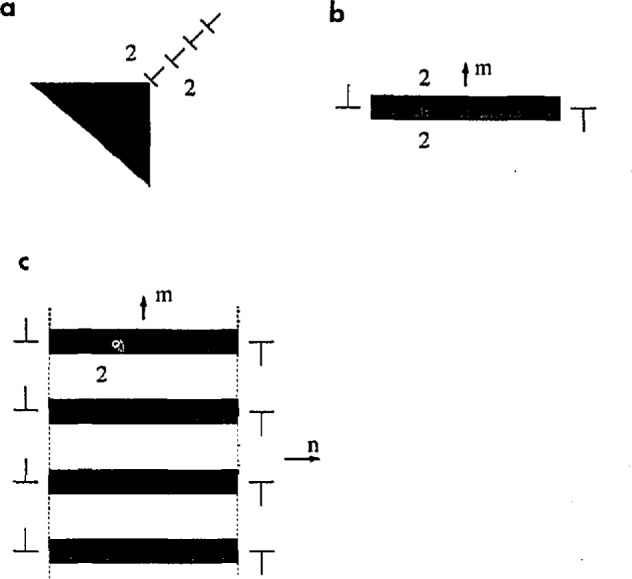
Schematic drawing of two rotational domains (white and gray shades) separated by planar SFIs and corresponding long range elastic stress fields, (a) Disclination field of a dihedral angle of a domain interface, (b) Dislocation-like field of a single domain inside another domain serving as a matrix, (c) Self-accommodated group of domains with reduced long range field.

Two rotational domains separated by a planar SFI are a unique morphology that avoids long range elastic stress fields. Two domains cannot be bounded by the two conjugate orthogonal SFIs as shown in Fig. 7a, because the corner where these SFIs intersect each other would be a disclination, and therefore a source of a long-range distortion.

The optimal shape of one domain included inside another is a plate with a small thickness to length ratio. (Experimentally the plates usually are found lenticular.) If the wide facets of the plate are SFIs, the stress field would be concentrated only near the plate edge, in a manner similar to a dislocation loop field (Fig. 7b) [35, 37]. This long range field can be reduced if a packet of plate-like domains is formed (Fig. 7c). If the boundaries of the packets (imaginary planes through the plate edges) are aligned parallel to the conjugate SFI plane ***n*** of the SFI plane of the individual plates, ***m***, then interference of the edge fields cancels the long-range stress field components. Such plane-parallel packets (also named in the literature as *polytwins* (by analogy with polysynthetic twins, or polydomains) are a typical element of domain morphology.

**Fig. 8:**
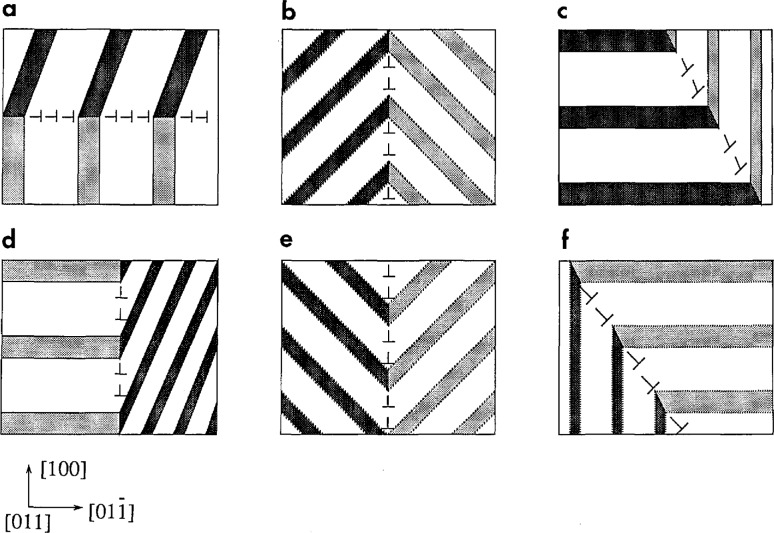
Polydomain structures of two second order polytwin plates composed of different combinations of three rotational variants (white, light and dark shades) of the orthorhombic phase as seen in the [011]c direction. The domain interfaces are either SFI (twin boundaries) or low-angle boundaries (dislocation walls). Continuous lines represent "edge-on" planes while doted lines are inclined planes. Possible combinations of three variants are: (a) (l,3)/(2/3), (2,4)/(l,4); (b) (4,5)/(2,5), (2,6)/4,6), (3,5)/(l,5), (l,6)/(3,6); (c) (l,2)/(2,4), (3,4)/(2,4), (1,2)/(1,3), (3,4)/(l,3); (d) (l,2)/(2,3), (1,2)/(1,4), (3,4)/(2,3), (3,4)/(l,4); (e) (4,6)/(2,6), (l,6)/(3,6), (1,5)/(3,5), (4,5)/(2,5); (f) (l=2)/(2,4), (3,4)/(l,3). (The labeling of the variants follows Fig. A.2.)

The polytwin as a whole can be considered as an effective “domain” of second order in a hierarchy of domain structures [33,37]. By analogy domain structures of even higher order can also be constructed. Examples of a domain structure of 2nd order for the BCC→Ort transition consisting of 3 variants are discussed below and illustrated in Fig. 8.

The pseudo-SFIs between polytwins can be determined by the same Eqs. (4) and (5), where *e_ij_* (or *S_ij_*) is an *average* self-strain of the polytwin as a whole. For example, the equations for the SFI between the polytwin consisting of domains 1 and 2 and the polytwin consisting of 1 and 3 is

$$x_{i}[e_{ij}(1,2)-e_{ij}(1,3)]x_{j}=0$$ (6)

The average self-distortions *S_ij_* (1,2) and *S_ij_* (1,3) are expressed through an average distortion of the polytwins

$$S_{ij}(1,2)=(1-\alpha )S_{ij}(1)+\alpha S_{ij}(2)$$ (7a)

$$S_{ij}(1,3)=(1-\beta )S_{ij}(1)+\beta S_{ij}(3)$$ (7b)

where *α* (or *β*) is the fraction of domains 2 (or 3) in polytwin (1,2) (or 1,3), and where the distortions *S_ij_*(1), *S_ij_*(2) and *S_ij_*(3) include the supplementary rotations [Eq. (3)] of the domains in the polytwins required for conserving coherency.

In general, to determine the pseudo-SFI between polytwins, the fractions *α* and *β* must be known. If *α* = *β*, the stress free boundary between polytwin (1,2) and polytwin (1,3) may run along the SFI between domains 2 and 3. For that, a line of intersection of the 1,2 SFI and the 1,3 SFI has to belong to the 2,3 SFI. For the BCC→ORT transformation there are three different interfaces of this type between polytwins consisting of 3 types of domains: along {100}_c_, {110}_c_ and {*h*11}_c_ according to the three possible orientations of the SFIs between the domains given in Table 4. Using Table 4 it is not difficult to find all possible second order polydomain morphologies for the *α* = *β* case. Such morphologies for three-variant structures are represented in Fig. 8. It is very likely that such structures with *α* = *β* correspond to a minimal energy.

While interfaces between polytwins that satisfy Eq. (6) have no long-range stress field, they do have microstresses distributed in the packet boundary. Even in the case of good matching {*α* = *β*), the rotation between different domains causes microstresses which can be described as fields from disclination dipoles. These microstresses at the boundary could manifest themselves during annealing as sites for further microstructural change.

The approach of packing first, second, and higher order effective domains can be applied in principle for the analysis of any hierarchy of domain structures. The scale of such hierarchical structures should be determined by the competition between the short-range microstresses distributed in the packet boundaries that tend to disperse the structure and the effective interfacial energy that tends to coarsen the structure.^3^ The number of the variants in the polydomain structure which are necessary to accommodate the self strain depends on boundary conditions. For a polydomain structure inside an untransformed matrix, the simplest polytwin that has an invariant plane boundary with the matrix is sufficient [39,40]. If the boundary of the region to be transformed is fixed, e.g., it coincides with a grain boundary, minimum elastic energy corresponds to the minimum average self-strain of the region, or zero average shear. This condition can be achieved only when all variants take part in the polydomain structure. The number of the variants determines an internal hierarchy of the polydomain structure.

## 5. Expected Microstructures and Transformation Paths

The present analysis suggests that three different types of domain structures are possible for a single phase microstructure of the O-phase depending on the transformation path traversed. Paths 1–3 are summarized in the form of subgroup sequences. These paths differ primarily as to whether the hexagonal symmetry phases or the B19 phase occurs at an intermediate stage of transition. The path involving the hexagonal phases (path 1) involves the formation of a supergroup; i.e., the intermediate orthorhombic A20 structure transforms to the hexagonal A3 by pure displacement. In general this is impossible (as a pure displacive transformation) if the parent phase has either long or short range chemical order of a type which would have been required to adjust to form the higher symmetry [41]. Thus path 1 is only possible for alloys quenched from a disordered BCC phase.

For Ti-Al-Nb alloys quenched from a B2 phase field, path 2 is clearly expected. The path is characterized by the presence of the B19 structure as an intermediate stage of transition, which in this case forms by a purely displacive transition from B2. The B19 phase could also form from an alloy quenched from the disordered BCC field by path 2 or by path 3. This latter path involves the formation of the B19 structure from the orthorhombic A20 by a pure ordering reaction between Ti and Al/Nb. Experimentally, evidence for the occurrence of one of the three paths can be obtained with microstructural information for the transient existence of B19 or A3 phases in the final O-phase domain structure. In alloys near the Ti_3_Al-Nb_3_Al section of the ternary system, the tendency towards B2 order in the high temperature BCC phase is strongest for alloys near Ti_2_AlNb because the two Wyckoff sites of the B2 are known [21] to be filled with Ti and a mixture of Al and Nb. Thus paths to the O-phase involving the B19 phase are most likely for alloys with Nb contents around 25 at%, while the path to the O-phase involving the hexagonal phase is expected for lower levels of Nb.

**Fig. 9:**
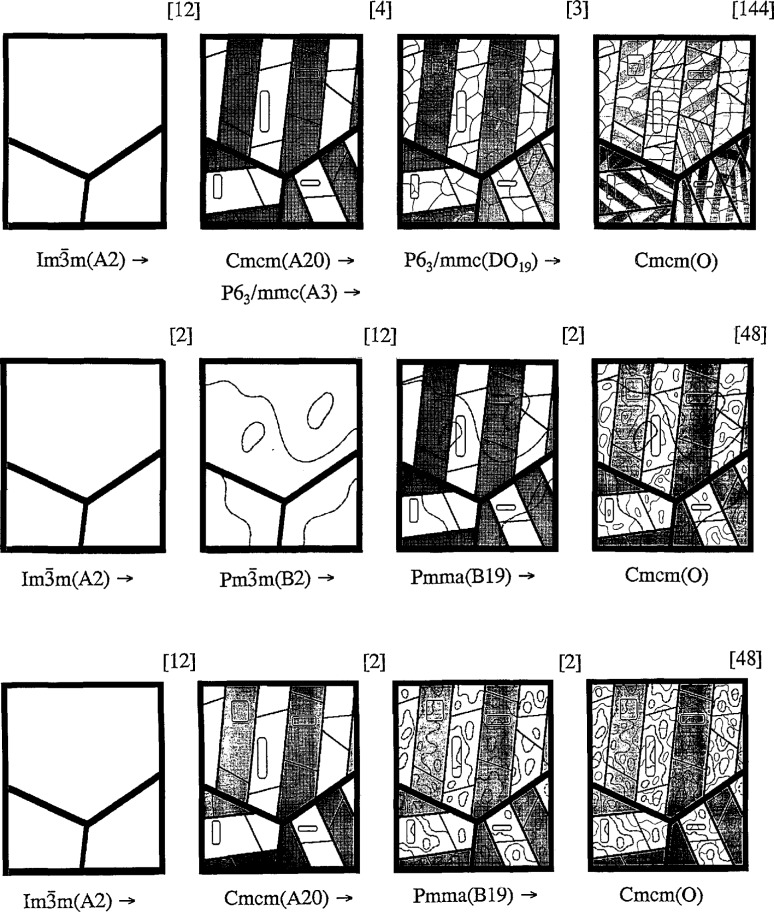
Graphical representation of the microstructural development for the three paths, starting from a large grain single phase BCC and ending with single phase orthorhombic. For the figure it is assumed that the interface configuration does not change significantly after formation at each stage of the transformation.

The microstructural development for the three paths is depicted in Fig. 9, starting from a large grain single phase BCC and ending with single phase orthorhombic. It is assumed that the interface configuration does not change significantly after formation at each stage of the transformation. All three paths, in their first stages, have similar microstructures composed of orthorhombic phase domains (either disordered Cmcm(A20) for paths 1 and 3 or ordered Pmma(B19) for path 2). According to the discussion in Sec. 4, the domains will form a polytwin morphology with SFIs parallel to either {100}_c_, {110}_c_ or conjugate (*hhk*)_c_ planes. In path 2, the BCC→B2 ordering precedes the formation of the orthorhombic phase but does not influence the formation and morphology of the polytwin structure. The APBs due to this ordering (curved lines) separate either interconnected or closed volume domains and may be found continuously crossing the polytwin domains. If path 2 starts from the B2 phase, such APBs will be absent.

Inside the polytwin plate-like domains, as Fig. 9 shows, anisotropic planar interfaces (schematically represented as rectangles, or straight lines for interfaces connected to twin boundaries) separate two translational domains resulting from antiphase shuffles (formally due to the Fmmm→Cmcm and Cmmm→Pmma symmetry changes). The anisotropy is expected because of the stacking fault nature of the interface structure. Because of the anisotropy the interfaces are distinct for each orthorhombic phase variant orientation.

After the formation of the polytwin structure by displacive ordering, the next step in all three transformation paths is chemical ordering. For path 1 the ordering involves two steps. First, A3→DO_19_ ordering of the hexagonal lattice (between Al and Ti/Nb) results in a four translational domain structure with isotropic interfaces shown in Fig. 9 as thin lines with triple junctions. Some of the interfaces are shown to coincide with previously formed translational interfaces. These coinciding segments will have a structure where changes in both atomic environment and distances are combined. Secondary ordering (DO_19_→O-phase) between Ti and Nb results in a second polytwin domain structure with planar interfaces running through the DO_19_ APBs, which are not effected by the secondary ordering. The interfaces in the same primary plate can have different orientations (either orthogonal or 60° rotated) as discussed in Appendix A and shown in Fig. 9.

In path 2, the ordering (B19→O-phase) between Al and Nb results in a two domain structure, with isotropic interconnected or closed interfaces. Due to the presumed lower temperature of transformation for this stage, the size of these antiphase domains is shown in Fig. 9 smaller then of those from the first BCC→B2 ordering. If the path starts from the B2 phase only the second type of antiphase domains will occur in the final microstructure.

In path 3 there are two steps of chemical ordering–the first one between Ti and Al/Nb atoms (A20→B19) and the second one between Al and Nb (B19→O-phase)–resulting in isotropic interfaces. Again, due to the difference in the presumed temperature of transformation, the size of these antiphase domains may be different. However the difference, as it is shown in Fig. 9, is less than for path 2, and this is the only difference in these two final microstructures.

## Figures and Tables

**Table 1:** Subgroups and supergroups from the International Tables of Crystallography for $Im\bar{3}m$ and P6_3_/mmc^a^

**(a)** $Im\bar{3}m$		
Maximal non-isomorphic subgroups		
	I	[3] I4/m 12/m (I4/mmm)
		[3] I4/m 12/m (I4/mmm)
		[3] I4/m 12/m (I4/mmm)
→		[4] $I1\bar{3}2/m(R\bar{3}m)$
		[4] $I1\bar{3}2/m\;(R\bar{3}m)$
		[4] $I1\bar{3}2/m\;(R\bar{3}m)$
		[4] $I1\bar{3}2/m\;(R\bar{3}m)$
		[2] Im3¯1(Im3¯)
		[2] 1432
		[2] $I\bar{4}3m$
→	IIa	[2] $\mathrm{Pm}\bar{3}m$
		[2] $\mathrm{Pn}\bar{3}n$
		[2] $\mathrm{Pm}\bar{3}n$
		[2] $\mathrm{Pn}\bar{3}m$
	IIb	none
Maximal isomorphic subgroups of lowest index		
	IIc	[27] Im3¯m (*a*′ = 3*a*, *b*′ = 3*b, c*′=3*c*)
Minimal non-isomorphic supergroups		
	I	none
	II	[4] $\mathrm{Pm}\bar{3}m$ (2*a*′=*a*, 2*b*′=*b*, 2*c*′=*c*)
**(b) P6_3_/mmc**		
Maximal non-isomorphic subgroups		
	I	[2] P6_3_222
		[2] P6_3_/m11(P6_3_/m)
		[2] P6_3_mc
		[2] $P\bar{3}\mathrm{ml}$
		[2] $P\bar{3}1c$
		[2] $P\bar{6}m2$
		[2] $P\bar{6}2c$
→		[3] Pmmc (Cmcm)
		[3] Pmmc (Cmcm)
		[3] Pmmc (Cmcm)
	Ila	none
	IIb	[3] H6_3_/mmc(*a*′ = 3*a*,*b*′ = 3*b*)(P6_3_/mcm)
Maximal isomorphic subgroups of lowest index		
→	IIc	[3] P6_3_/mmc(*c*′ = 3*c*;[4]P6_3_/mmc(*a*′ = 2a,*b*′=2*b*)
Minimal non-isomorphic subgroups		
	I	none

a (a) the Im3¯m space group of the BCC structure and (b) the P6_3_/mmc (of both simple HCP and ordered DO_19_) hexagonal structures. Different types of subgroups are listed according to: 1–no change of translations; IIa–decentering; IIb–enlarging the conventional cell; IIc–no change of the group type, [x]–index of the subgroup which gives the number of variants.

**Table 2:** Description of various phases based on common sites in the Cmcm space group

Structure	Lattice Conditions	Occup.	Wyck.	*x*	*y*	*z*
Ti_2_AlNb, HgNa, Cmcm	*y*_1_ =0.163; *y*_2_=0.623	A	4c_1_	0	*y*_1_	1/4
	*y*_3_ = 0.904; *x*_3_ = 0.231	B	4c_2_	0	*y*_2_	1/4
		C	8g	*x*_3_	*y*_3_	1/4
Ti_3_Al, DO_19_	$b/a=\sqrt{3}$	A	4c_1_	0	1/6	1/4
Ni_3_Sn, P6_3_/mmc		B	4c_2_	0	2/3	1/4
		B	8g	1/4	11/12	1/4
αTi,A3	$b/a=\sqrt{3}$	A	4c_1_	0	1/6	1/4
Mg, P6_3_/mmc		A	4c_2_	0	2/3	1/4
		A	8g	1/4	11/12	1/4
Ti-Nb, A20	*y*_1_ = 0.1	A	4c_1_	0	*y*_1_	1/4
αU, Cmcm		A	4c_2_	0	1/2+ *y*_1_	1/4
		A	8g	1/4	3/4+ *y*_1_	1/4
Ti-Ni, B19	*y*_1_= 0.156	A	4c_1_	0	*y*_1_	1/4
AuCd, Pmam(Pmma)	*y*_2_= 0.906	A	4c_2_	0	1/2*+ y*_1_	1/4
		B	8g	1/4	*y*_2_	1/4
βTi, A2	$b/a=\sqrt{2}$	A	4c_1_	0	1/8	1/4
W, Im3¯m	$c/a=\sqrt{2/2}$	A	4c_2_	0	5/8	1/4
		A	8g	1/4	7/8	1/4
TiNi, B2	$b/a=\sqrt{2}$	A	4c,	0	1/8	1/4
CsCl, $\mathrm{Pm}\bar{3}m$	$c/a=\sqrt{2/2}$	A	4c_2_	0	5/8	1/4
		B	8g	1/4	7/8	1/4

**Table 3:** List of interfaces between domains in different group/subgroup transitions. The Class represents type of symmetry reduction [37], the interfaces are described by domain generating symmetry operation (of lowest symmetry)

Group/subgroup	Class of subgroup	Type of interface
Im3¯m→ $\mathrm{Pm}\bar{3}m$	IIa	translational (APB)
Im3¯m→Cmcm(A20)	I+I + IIa	rotational (twins of I and II kind), translational with stacking fault mixed twin/translational
Cmcm(A20)→P6_3_/mmc(A3)	Supergroup	no new interface
P6_3_/mmc(A3) →P6_3_/mmc(DO_19_)	IIc	translational (APB)
P6_3_/mmc(DO_19_)-»Cmcm(O)	I	rotational (compound twins)
$\mathrm{Pm}\bar{3}m$→Pmma(B19)	I + I+IIa	rotational (twins of I and II kind), translational with stacking fault, mixed twin/translational
Cmcm(A20)→Pnima(B19)	IIa	translational (APB)
Pmma(B19)→Ctncm(0)	IIb	translational (APB)

**Table 4:** List of SFI interfaces for all possible pairs of domain of the O-phase in the $m\bar{3}m\to \mathrm{mmm}$ type transformation. Labeling of variants and interface indexes are given in the cubic coordinates of Fig. A.2. *s = k/h* =2B/(*A – C*)

Pair of domains	Interface equation	Inrerface in Miller indices
1/2	*x* = 0; *y*=0;	(1 0 0) (0 1 0)
1/3	*y* = z; *−*2*Bx +* (*C−A*)(*y+z*) = 0;	(0 1 1) (*s* 1 1)
1/4	*y = −z;* *−*2*Bx +* (*C−A*)(*y−z)* = 0;	(0 1 1) (*s* 1 $\bar{1}$)
1/5	*x = z;* *−*2*By +* (*C−A*)(*x+z*) = 0;	(1 0 $\bar{1}$) (1 *s* 1)
1/6	*x = −z;* *−*2*By* + (C*−A*)(*x−z*) = 0;	(1 0 1) (1 *s* $\bar{1}$)
2/3	*y = −z*; 2*Bx +* (*C−A*)(*y−z*) = 0;	(0 1 1) (*s* $\bar{1}$ 1)
2/4	*y =z*; 2*Bx +* (*C−A*)(*y+z*) = 0;	(0 1 $\bar{1}$) (*s* $\bar{1}$ $\bar{1}$)
2/5	x = *− z*; 2*By +* (*C−A*)(*y−z*) = 0;	(1 0 1) ( $\bar{1}$ *s* 1)
2/6	*x = z;* 2*By +* (*C−A*)(*y+z*) = 0;	(1 0 $\bar{1}$) ( $\bar{1}$ *s* $\bar{1}$)
3/4	*x* = 0; *z* = 0;	(1 0 0) (0 0 1)
3/5	*x =y;* *−*2*Bz +* (*C−A*)(*x+y*) = 0;	(1 $\bar{1}$ 1) (1 1 *s*)
3/6	*x = −y*; *−*2*Bz +* (*C−A*)(*x−y*) = 0;	(1 1 0) (1 $\bar{1}$ *s*)
4/5	*x = −y*; 2*Bz +* (*C−A*)(*x−y*) = 0;	(1 1 0) ( $\bar{1}$ 1 *s*)
4/6	*x = −y*; 2*Bz +* (*C−A*)(*x+y*) = 0;	(1 $\bar{1}$ 0) ( $\bar{1}$ $\bar{1}$ *s*)
5/6	*y* = 0; *z* = 0;	(0 1 0) (0 0 1)

**Fig. A.1:**
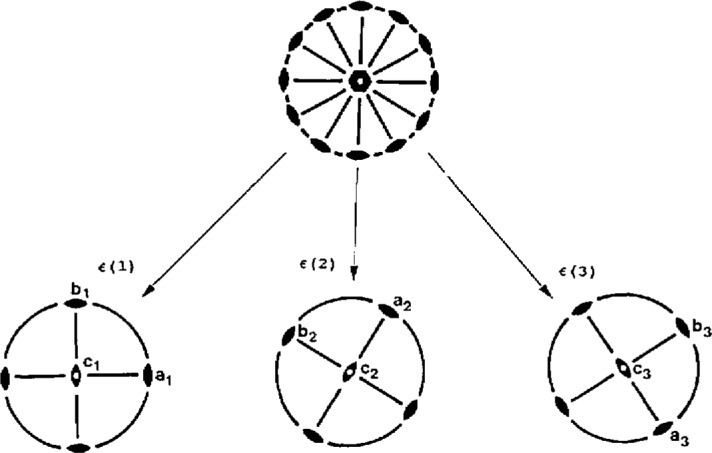
Stereographic projections of the point groups of the hexagonal (6/mmm) and of the orthorhombic phase variants (mmm’s), according to their lattice correspondence. When the symmetry elements of a pair of variants are compared, a set of two orthogonal mirror planes is found that belong to the parent phase but not to the pair considered.

**Fig. A.3:**
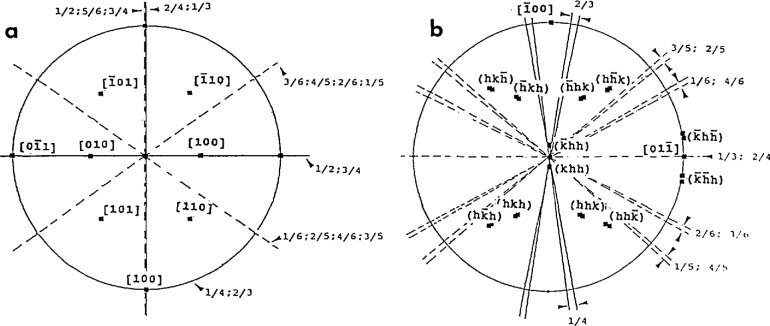
Directions of the SFI traces superimposed on the [011]_c_ stereographic projections, as they would be seen for crystals oriented for TEM at the [011]_c_ zone axes, (a) traces of the symmetric {001}- and {110}-type interfaces, (b) traces of the non-symmetric lattice parameter depended {*hhk*}-type interfaces. A range of possible orientations for the orthorhombic phase having lattice parameters ranging from those of the Ti_2_AlNb O-phase (determined in [24] as *a* =0.60893 nm, *b* =0.95594 nm, *c* =0.46666 nm) to those of the hexagonal DO_19_ phase (determined in [4] as *a* = 0.578 nm, *b* = 1.001 nm, *c* = 0.646 nm) is shown as shaded area.

## Footnotes

1 A subgroup ***H*** of space group ***G*** is called a maximal subgroup of ***G*** if there is no subgroup ***L*** of ***G*** such that ***H*** is a subgroup of ***L***, i.c., ***G***>***L***>***H***[**27]**

2 The use of an intersection group to create continuity of group/subgroup relations has been successfully used in a study of the formation of *ω*-type phases from a B2 high-temperature phase in a different region of the Ti-Al-Nb system containing 37.5 at% Al and 12.5 at% Nb [32]. In this case the [111](111) shuffle displacement wave of the omega transition has the cylindrical ∞h symmetry (for coinciding wave and polarization vectors) [30]. The intersection point group of the $m\bar{3}m$ (BCC) and the ∞h (with the mirror plane h parallel to one of the 〈111〉_c_ directions) has the trigonal $\bar{3}m$ point group symmetry. The $P\bar{3}m1$ space group ( $\bar{3}m$ point group) is obtained from the intersection of space groups of the two stable phases, the high-temperature B2 ( $\mathrm{Pm}\bar{3}m$) and the low-temperature hexagonal B8_2_ (P6_3_/mmc). The structure with the trigonal $P\bar{3}\mathrm{ml}$ symmetry was indeed observed as an intermediate state in the transformation path.

3 As shown by [33,35,37], similar hierarchical structures can also be formed by domains consisting of different phases. For example, the structure morphologically similar to the 3-domain structure in Fig. 8 can be created by incorporating two domains of the O-phase inside of which there is one domain of the hexagonal DO_19_ phase (domain 1). The geometry of such a heterophase structure will be analyzed in Bendersky and Boettinger [J. Res. Natl. Inst. Stand. Technol. 98, 585 (1993).

## 6. Appendix A. SFIs for the P6_3_/mmc (DO_19_)→Cmcm(O) (6/mmm→mmm) Transition

The structural relation between the phases (o – orthorhombic; h–hexagonal) gives the following lattice correspondence: ***a***_0_ = ***a***_1h_; ***b***_0_ = ***a***_1h_ + 2***a***_2h_; ***c***_0_ = ***c***_h_. Fig. A.1 shows stereographic projections of the point groups of the hexagonal and of the orthorhombic phase variants, according to the lattice correspondence. When the symmetry elements of a pair of variants are compared, we find a set of two orthogonal mirror planes which belong to the parent phase but not to the pair considered, e.g., for variants 2 and 3 in Fig. A.1, the set of lost mirror planes is *x* =0 and *y* =0. Similarly, for the 1/2 and l/3 pairs of domains the sets are $y/x=\tan\;30{}^{\circ}=\sqrt{3/3}$, $y/x=\tan\;120{}^{\circ}=-\sqrt{3}$ and $y/x=\tan\;60{}^{\circ}=\sqrt{3}$, $y/x=\tan\;150{}^{\circ}=-\sqrt{3/3}$, respectively. Being mirror planes, and therefore twinning planes, the three sets are planar SFIs running parallel to the z-axis.

The same results can be obtained by solving Eq. (4). For this purpose, the self-strain tensor of the three variants must be first determined. For variant 1 in Fig. A.1, the self-strain tensor will be

$$e_{ij}(1)=\left|\begin{array}{ccc} e_{1} & 0 & 0\\ 0 & e_{2} & 0\\ 0 & 0 & e_{3} \end{array}\right|,$$ (A.1)

where

$$e_{1}=a+a^{2}/2;\;e_{2}=b+b^{2}/2;\;e_{3}=c+c^{2}/2.$$

with

$$a=\frac{a_{0}-a_{h}}{a_{h}};b=\frac{b_{0}-\sqrt{3}a_{h}}{\sqrt{3}a_{h}};c=\frac{c_{0}-c_{h}}{c_{h}}.$$

The self-strain tensor for the other two variants will be obtained using Eq. (1). The variant generating symmetry operation in that case is chosen to be the three-fold anti-clock-wise rotation (*g_ij_* =3^−^), so that *e_ij_* (3) = 3^−^3^−^*e_ij_* (1) and *e_ij_* (2) = 3^−^3^−^ *e_ij_*(3) (see Fig. A.1). In order to do calculations in orthogonal coordinates, the matrix of the three-fold rotation must be presented in the same coordinates. It is found as

$$3_{ij}^{-}=\left|\begin{array}{ccc} -1/2 & -\sqrt{3}/2 & 0\\ \sqrt{3}/2 & -1/2 & 0\\ 0 & 0 & 1 \end{array}\right|.$$ (A.2)

After matrix multiplication we find that

$$e_{ij}(3)=\left|\begin{array}{ccc} \frac{a}{4}+3\frac{b}{4} & -\sqrt{3}\frac{a}{4}+\sqrt{3}\frac{b}{4} & 0\\ -\sqrt{3}\frac{a}{4}+\sqrt{3}\frac{b}{4} & 3\frac{a}{4}+\frac{b}{4} & 0\\ 0 & 0 & c \end{array}\right|,$$ (A.3)

and

$$e_{ij}(3)-e_{ij}(1)=\frac{\sqrt{3}(b-a)}{4}\left|\begin{array}{ccc} \sqrt{3} & 1 & 0\\ 1 & -\sqrt{3} & 0\\ 0 & 0 & 0 \end{array}\right|.$$ (A.4)

Equation (4) for the SI interfaces between variants 3 and 1 will be

$$[e_{ij}(3)-e_{ij}(1)]x_{i}x_{j}=x^{2}-y^{2}+\frac{2}{\sqrt{3}}xy=0$$ (A.5)

or

$$(y-\sqrt{3}x)(y+x/\sqrt{3})=0.$$

Two solutions for the quadratic equation are $y/x=\sqrt{3}$ and $y/x=-\sqrt{3}/3$ and they do not depend on the parameters *a, b*, and c. These are the same mirror planes found with the help of the stereographic analysis performed above. Solutions for the other variants can be found similarly, and they are the remaining mirror planes of the 6/mmm with $y/x=\sqrt{3}/3,-\sqrt{3}$ (for the 1/2 variants) and *x*=0, *y* = 0 (for the 2/3 variants). A pair of variants can be identified unambiguously from their interface orientation.

## 7. Appendix B. SFIs for the Im3¯m(A2) →Cmcm(A20) and $\mathrm{Pm}\bar{3}m$(B2) →Pmma(B19) ( $m\bar{3}m$→mmm) Transition

The structural relation between the phases (c–cubic; o – orthorhombic) gives the following lattice correspondence: ***a*_0_=*a*_1c_; *b*_0_=*a*_2c_+*a*_3c_; *c*_0_=*a*_2c_−*a*_3c_** According to the lattice correspondence, Fig. A.2 shows the stereographic projections of the point groups and the crystallographic axes of the parent cubic ( $m\bar{3}m$) and its six subgroup variants of the orthorhombic (mmm) phases. Comparing symmetry elements belonging to a pair of the variants with those of the parent cubic, we can find mirror planes of the parent (but missing in the product variants) that reflect the variants into each other. The mirror planes serve as the SFIs. For a pair of variants sharing the *a*_0_ axis (1/2, 3/4, and 5/6 pairs in Fig. A.2), there are two orthogonal mirror planes of {100}*_c_* type (parallel to the *a*_0_ axis). For the other pair of variants, not sharing a common direction, there is only one such mirror plane, of {110}*_c_* type. Orthogonal to the {110} plane is either a ${\left\{h\bar{h}k\right\}}_{c}$ or a ${\left\{h\bar{h}\bar{k}\right\}}_{c}$ plane, generally of an irrational orientation which depends on the ratio of the lattice parameters. In order to find *h* and *k*, Eq. (4) must be solved.

We solve Eq. (4) in the coordinate system of the cubic phase shown in Fig. A.2. First, we find the self-strain tensors of the six variants using the variant generating operators, Eq. (1). All variants can be generated by the mirror plane operations, starting from the first variant:

$$\begin{array}{l} e(2)=m_{100}m_{100}e(1),\;e(5)=m_{10-1}m_{10-1}e(1),\\ e(4)=m_{011}m_{011}e(1),\;e(6)=m_{010}m_{010}e(5),\\ e(3)=m_{100}m_{100}e(4). \end{array}$$ (B.l)

Matrices for the mirror planes are given in Table 11.4 of *The International Tables for Crystallography* [37]. The self-strain tensor for variant 1 (see Fig. A.2) in the coordinates of the variant is

$$e_{i^{\prime }j}(1)=\left|\begin{array}{ccc} a & 0 & 0\\ 0 & b & 0\\ 0 & 0 & c \end{array}\right|,$$ (B.2)

where

$$a=\frac{a_{0}-a_{c}}{a_{c}},\;b=\frac{b_{0}-\sqrt{2}a_{c}}{\sqrt{2}a_{c}},\;c=\frac{c_{0}-\sqrt{2}a_{c}}{\sqrt{2}a_{c}};$$

and *a_c_*, *a*_0_, *b*_0_,* c*_0_ are the lattice parameters of the cubic and the disordered orthorhombic phase (for the ordered O-phase half of their values of *a*_0_ and *b*_0_ should be used).

In order to obtain the $e_{i^{\prime }j}(1)$ tensor in cubic coordinates, the tensor’s axes must be rotated 45° around [100]_c_, which is obtained by the rotation and permutation matrix

$$\alpha_{ij}=\left|\begin{array}{ccc} 0 & m & m\\ 0 & -m & m\\ 1 & 0 & 0 \end{array}\right|,m=\frac{\sqrt{2}}{2}.$$ (B.3)

The tensor *e_ij_*(1) in cubic coordinates is then

$$e_{ij}(1)=\alpha_{ik}\alpha_{jl}e_{k^{\prime }l}(1)=\left|\begin{array}{ccc} A & B & 0\\ B & A & 0\\ 0 & 0 & C \end{array}\right|,$$ (B.4)

where *A* =1/2(*b*+c); *B* = 1/2(*c*−*b*); *C*=*a*.

The self-strain tensors for other variants are:

$$e_{ij}(2)=\left|\begin{array}{ccc} A & -B & 0\\ -B & A & 0\\ 0 & 0 & C \end{array}\right|,e_{ij}(3)=\left|\begin{array}{ccc} A & 0 & B\\ 0 & C & 0\\ B & 0 & A \end{array}\right|;$$ (B.5)

$$e_{ij}(4)=\left|\begin{array}{ccc} A & 0 & -B\\ 0 & C & 0\\ -B & 0 & A \end{array}\right|,e_{ij}(5)=\left|\begin{array}{ccc} C & 0 & 0\\ 0 & A & B\\ 0 & B & A \end{array}\right|;$$ (B.6)

$$e_{ij}(6)=\left|\begin{array}{ccc} C & 0 & 0\\ 0 & A & -B\\ 0 & -B & A \end{array}\right|.$$ (B.7)

Equation (4) for the SFI interface between variants 1 and 2 will be

$$[e_{ij}(2)-e_{ij}(1)]x_{i}x_{j}=\left|\begin{array}{ccc} 0 & 2B & 0\\ 2B & 0 & 0\\ 0 & 0 & 0 \end{array}\right|x_{i}x_{j}=xy=0.$$ (B.8)

Solutions are the symmetric *x* = 0, (100)_c_, and *y* =0, (101)_c_, the twinning mirror planes expected from symmetry.

For variants 1 and 3 Eq. (4) is

$$[e_{ij}(3)-e_{ij}(1)]x_{i}x_{j}=\left|\begin{array}{ccc} 0 & B & -B\\ B & A-C & 0\\ -B & 0 & C-A \end{array}\right|x_{i}x_{j}=(y-z)[2Bx+(A-C)(y+z)].$$ (B.9)

One solution of the equation is the expected syrnmetric case, *y* =*z*, which corresponds to the ${\left(01\bar{1}\right)}_{c}$ crystallographic plane. The second, non-symmetric case, is 2*Bx +* (*A*−*C*)(*y*+z) = 0. This is the equation of a plane having a normal ***n*** where *n_x_* = 2*B*, ***n****_y_* = (*A*−*C*), and *n_z_* = (*A*−*C*). This plane has (*hkk*)_c_ Miller indexes, and therefore orthogonal to the ${\left(01\bar{1}\right)}_{c}$. The ratio of *h* to *k* is 2*B*/(*A*−*C*), or 2(*c*−*b*)/(*b+c*−2*a*) and depends only on the lattice parameters of the orthorhombic phases $\left[h/k=2(2c_{0}-b_{0})/(b_{0}+2c_{0}-2\sqrt{2a_{0}})\right]$. For the lattice parameters of the Ti-Al-Nb DO_19_ and O phases [4], the Miller indexes are close to ${\left\{\bar{1}55\right\}}_{c}$ and ${\left\{\bar{1}44\right\}}_{c}$, respectively.

Similarly, the solutions for all 15 pairs of domains were found. The results are given in Table 4. There are 30 (*N* = 6*×*5) SFIs, of which only 21 are different orientations: 3 of the {100}_c_ type, 6 of the {110}_c_ type and 12 of the {*hkk*}_c_ type.

Figure A.3 shows the directions of the SFI traces superimposed on the [011]_c_ stereographic projection, as they would be seen for crystals oriented for TEM at the [011]_c_ zone axis. Such drawings are useful in analyzing the nature of interfaces observed in TEM specimens of the Ti-Al-Nb alloys, as shown by Bendersky and Boettinger. Figure A.3a presents traces of the symmetric {001}_c_- and {110}_c_-type interfaces. Figure A.3b presents traces of the non-symmetric {*hhk*}_c_-type interfaces. Two of the {*hhk*}_c_-type interfaces are oriented edge-on ( $kh\bar{h}$ and $k\bar{h}h$), and two are inclined but with a rational trace direction $\left[01\bar{1}\right]$ (*khh* and $\bar{k}hh$). Since the lattice parameter varies with temperature and composition, a range of possible orientations of the interfaces is given for the orthorhombic phase having lattice parameters ranging from those of the Ti_2_AlNb O-phase (determined in [24] as *a*_0_ = 0.60893 nm, *b*_0_ = 0.95694 nm, *c*_0_ = 0.46666 nm) to those of the hexagonal DO_19_ phase (determined in [4] as *a*_h_ = 0.578 nm, *b*_h_ = 1.001 nm, *c*_h_ = 0.466 nm).
